# Rapid growth of mitotically active cellular fibroma of the ovary: a case report and review of the literature

**DOI:** 10.1186/s13000-016-0554-7

**Published:** 2016-10-22

**Authors:** Katsuya Matsuda, Seiko Tateishi, Yuko Akazawa, Akira Kinoshita, Shiko Yoshida, Sachiko Morisaki, Ai Fukushima, Takahiro Matsuwaki, Koh-Ichiro Yoshiura, Masahiro Nakashima

**Affiliations:** 1 Departments of Tumor and Diagnostic Pathology, Atomic Bomb Disease Institute, Nagasaki University, 1-12-4 Sakamoto, Nagasaki, 852-8523 Japan; 2 Department of Obstetrics and Gynecology, Japan Community Health Care Organization, Isahaya General Hospital, Isahaya, Japan; 3 Department of Gastroenterology and Hepatology, Nagasaki University Hospital, Nagasaki, Japan; 4 Department of Human Genetics, Atomic Bomb Disease Institute, Nagasaki University, Nagasaki, Japan

**Keywords:** Case report, Mitotically active cellular fibroma, Preoperative diagnosis, Rapid growth, *FOXL2* analysis

## Abstract

**Background:**

Mitotically active cellular fibroma (MACF) of the ovary, characterized by relatively high mitotic activity without severe atypia, represents a relatively new disease entity. MACF is categorized as a benign ovarian tumor. However, due to a limited number of case reports, its clinical and pathological features and optimum management remains largely undetermined. Herein, we report on a rare case of MACF that grew rapidly in size and was diagnosed on detailed pathological examination.

**Case presentation:**

A 44-year-old Japanese woman, who detected a myoma-like lesion 1-year earlier, was referred to our hospital when the follow-up examination demonstrated that the mass had increased in size. Magnetic resonance imaging revealed a T1 isointense and T2 hyperintense tumor (11 cm in diameter) in the right pelvic cavity. Laparoscopy confirmed the presence of a right ovarian tumor and laparoscopic right adnexectomy was performed. The tumor cells consisted of dense cellular proliferations of spindle fibroblast-like cells without significant cytological atypia. The mitotic activity index was estimated at >15 mitotic figures per 10 high-power fields. Reticulin staining and *FOXL2* mutation analysis excluded the possibility of an adult granulosa cell tumor, and the patient was diagnosed with a MACF of the ovary.

**Conclusions:**

To the best of our knowledge, we are the first to report on a case of rapid growth of a MACF of the ovary during follow-up. When an increase in the size of a solid ovarian mass is detected, a MACF should be considered as a differential diagnosis.

## Background

Ovarian fibrous tumors were previously classified as benign fibromas or malignant fibrosarcomas, according to the World Health Organization’s classification of tumors of the breast & Female Genital Origans [1]. Ovarian cellular fibromas were defined as having a mitotic figure of <3 per 10 high-power fields without severe nuclear atypia, and fibrosarcomas were defined as having a mitotic figure of ≥4 per 10 high-power fields with severe nuclear atypia [1]. Generally, the prognosis of patients with fibrosarcomas is extremely poor [2]. However, there have been a minority of cases where the patient was categorized as having fibrosarcoma with mild nuclear atypia, despite a high mitotic activity. These patients were associated with a relatively favorable prognosis [3–8]. In 2006, Irving et al. [9] defined these types of tumors as mitotically active cellular fibromas (MACFs), thus, distinguishing them from fibrosarcomas. Accordingly, MACFs have now been included under the heading of fibromas in the 2014 World Health Organization classification system [10]. MACFs are defined as having a mitotic figure of <3 per 10 high-power fields without severe nuclear atypia and fibrosarcomas are defined as having a mitotic figure of ≥4 per 10 high-power fields with severe nuclear atypia [10]. Several case reports have been published since MACFs were defined [11–16]. MACFs are associated with a more favorable prognosis than fibrosarcomas, with limited data on long-term survival rates available [17]. However, the natural history of MACFs is still largely unknown and suitable guidelines for diagnosing and treating this condition are lacking.

Herein, we report on the clinical and histopathological characteristics of a rare case of rapid growth of a MACF of the ovary that almost doubled in size during a 1-year follow-up period and provide a review of the literature.

## Case presentation

**Fig. 1:**
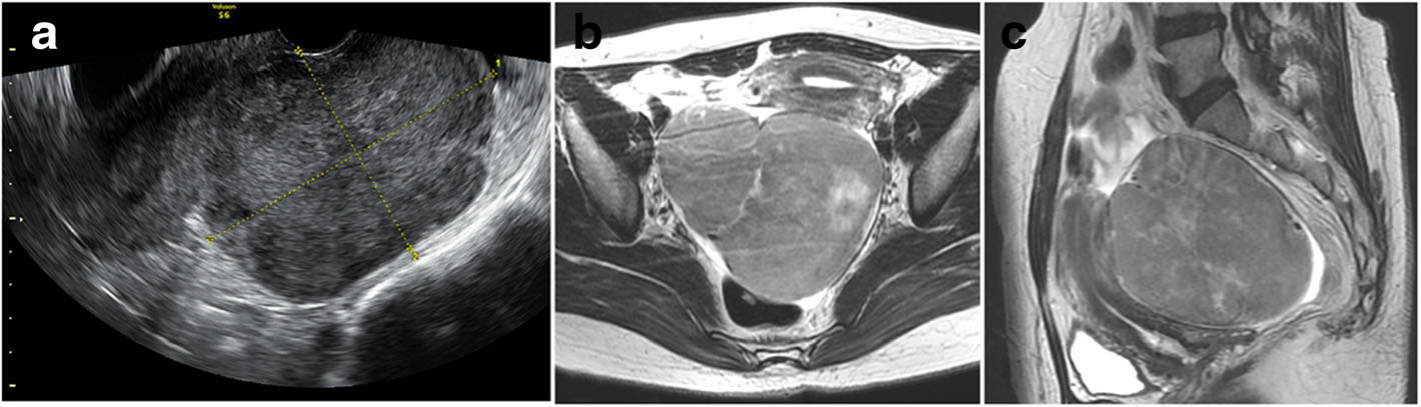
Preoperative transvaginal ultrasonography (**a**) detected an isoechoic solid mass in the right pelvic cavity (approximately 10 cm in size) with an ill-defined boundary between the lesion and the uterus, and T2-weighted magnetic resonance imaging of the ovarian tumor in (**b**) the horizontal and (**c**) the sagittal planes

**Fig. 2:**
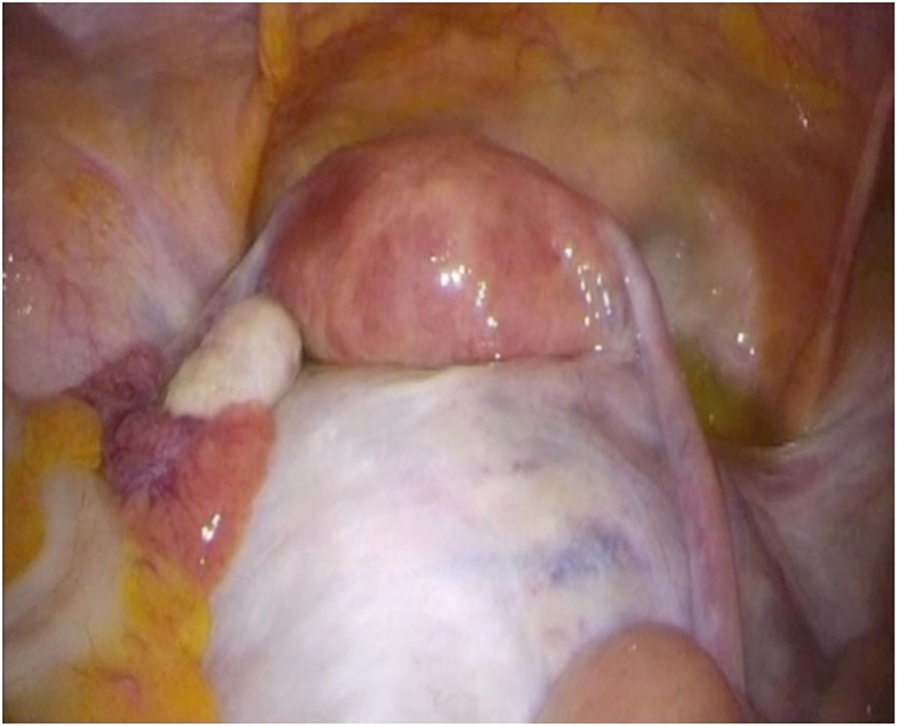
Laparoscopic findings. The uterus was of a normal size and the mass was a right ovarian tumor

A 44-year-old Japanese woman underwent a medical examination for health check reasons at her local hospital. An ultrasound scan revealed a uterine myoma-like lesion, 5.9 cm in diameter. The patient revisited the hospital 12-months later for a follow-up examination. In that time, the lesion had increased to approximately twice its size. The patient was referred to our clinic for a detailed examination. At the first internal examination, the body of the uterus was enlarged to the size of a newborn head; cervical and vaginal discharge was unremarkable. The adnexa were not palpable on both sides. The patient’s blood test results were normal. Transvaginal ultrasonography detected an isoechoic solid mass with an ill-defined boundary between the lesion and the uterus (Fig. 1a). A tumor, 110 × 90 × 80 mm in size, was revealed in the right pelvic cavity by magnetic resonance imaging. The tumor exhibited a slightly lobular pattern with smooth margins. The internal mass had a density similar to that of myometrium on abdominal, T1-weighted magnetic resonance imaging and a low density on T2-weighted magnetic resonance imaging in the horizontal plane (Fig. 1b). Several flow void regions (a bridging vascular sign) were detected between the lesion and the uterus. These findings suggested a subserous myoma. However, the continuity of the lesion and the uterus was unclear. Since we were unable to identify the right ovary, a fibroma/thecoma of the right ovary was considered as a differential diagnosis (Fig. 1c). The left ovary did not exhibit any abnormalities. At this point, a preliminary clinical diagnosis of subserous myoma was made and a laparoscopic myomectomy was planned. The laparoscopic findings demonstrated a 10-cm right ovarian mass without intra-abdominal adhesion or rupture of the tumor. The uterus and left appendages were normal in size (Fig. 2). A laparoscopic right adnexectomy was subsequently performed. The ascitic fluid was serous, and the cytological evaluation was negative.

**Fig. 3:**
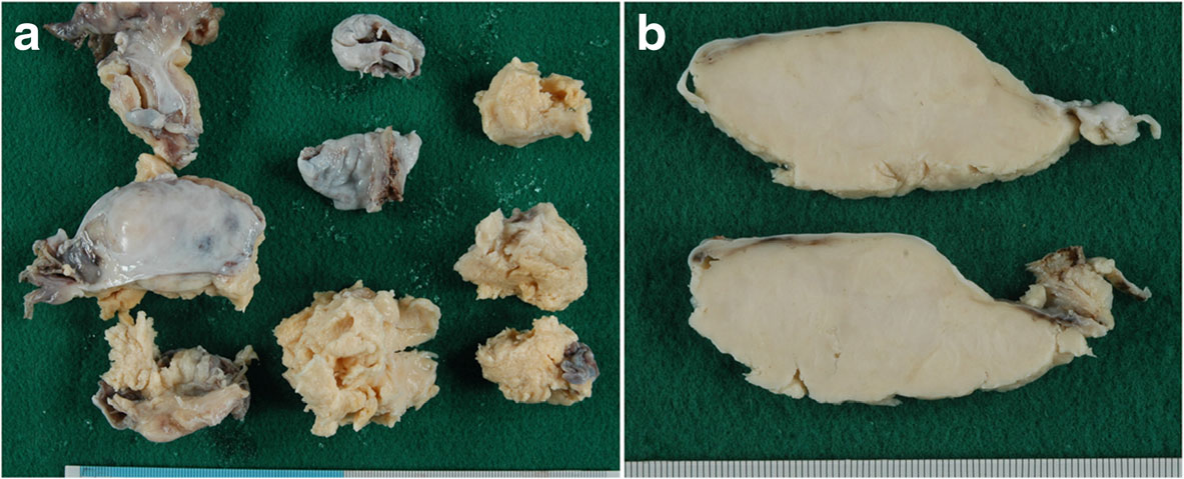
Macroscopic image of the right ovarian tumor. The cut surface of the tumor was homogeneous and solid in appearance with a yellowish-white pigment

**Fig. 4:**
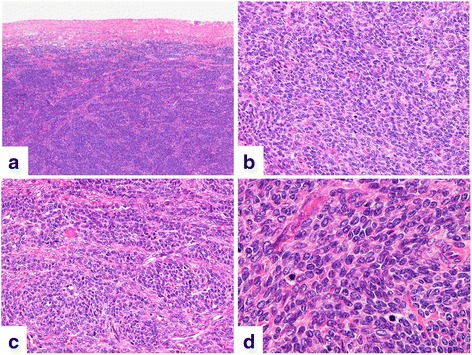
Histopathological findings of the right ovarian tumor. **a** The capsule of the tumor was intact without necrosis and hemorrhage; (**b**) dense cellular proliferations of spindle fibroblast-like cells, arranged in a fascicular structure with ovoid to spindle-shaped nuclei and a scant cytoplasm, and (**c**) tumor cell proliferations in a trabecular pattern with a collagen band distributed around the stroma; and (**d**) absence of significant cytological atypia. The mitotic activity index was estimated at >15 mitotic figures per 10 high-power fields (hematoxylin and eosin staining; original magnification [**a**] × 20, [**b**, **c**] × 100, and [**d**] × 400)

Macroscopically, the cut surface of the tumor was homogeneous and solid in appearance with a yellowish-white pigment. Necrosis and hemorrhage were absent within the tumor (Fig. 3a, b). Microscopic images are depicted in Fig. 4a–d. The capsule of the tumor was intact without necrosis and hemorrhage (Fig. 4a). The tumor cells consisted of dense cellular proliferations of spindle fibroblast-like cells, arranged in a fascicular structure, without significant cytological atypia (Fig. 4b). In addition, a portion of the tumor cells proliferated in a trabecular pattern and a collagen band was distributed around the stroma (Fig. 4c). The tumor was composed of cells with ovoid to spindle-shaped nuclei and a scant cytoplasm. Some of the nuclei had a nuclear groove. No severe cytological atypia was detected. The mitotic activity index was estimated at >15 mitotic figures per 10 high-power fields (Fig. 4d). Pathological differential diagnoses included a cellular fibroma, fibrosarcoma and adult granulosa cell tumor (AGCT).

**Fig. 5:**
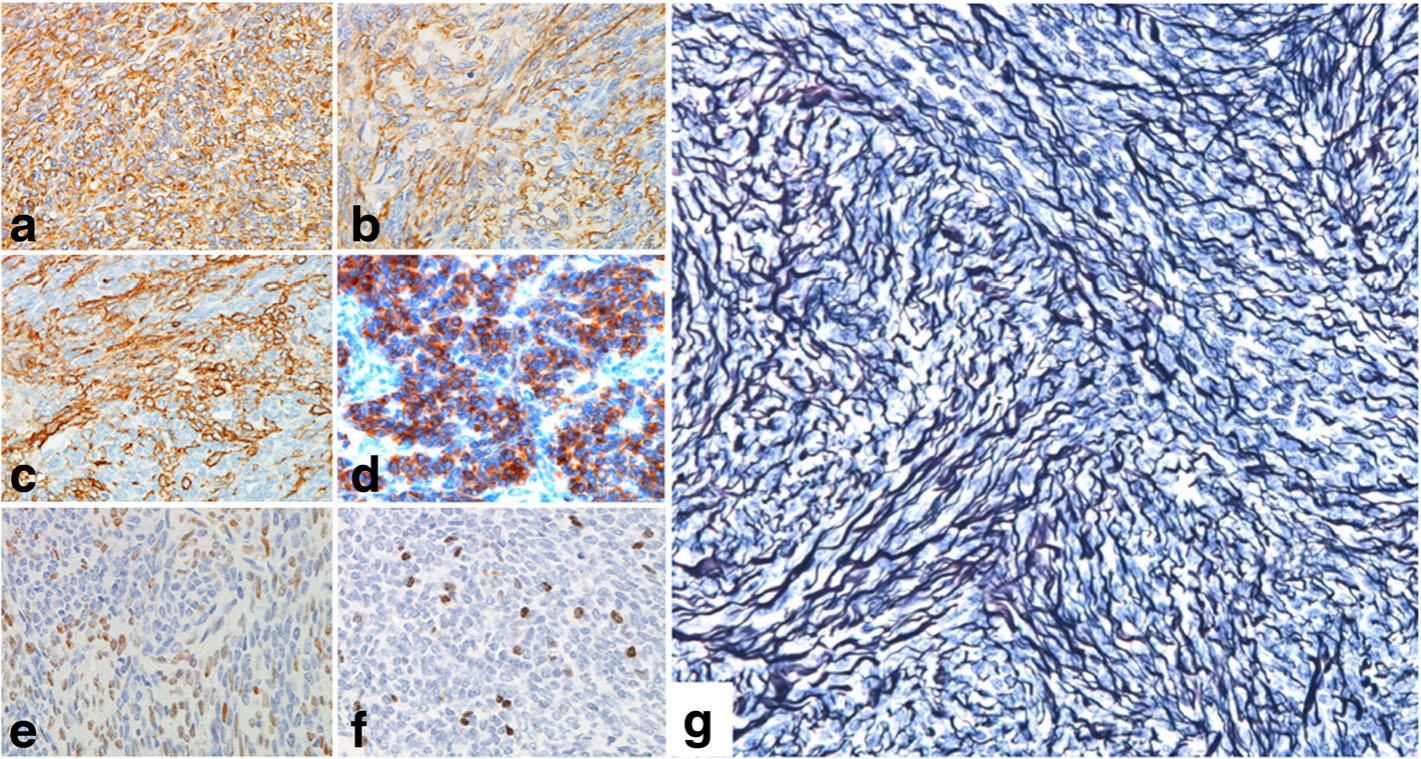
Immunohistochemical analysis of the right ovarian tumor exhibiting positivity for (**a**) vimentin, (**b**) muscle-specific actin, (**c**) alpha-smooth muscle actin, (**d**) alpha-inhibin and (**e**) estrogen receptor. **f** The Ki-67/MIB-1 labeling index was 9.5 % (original magnification × 200). **g** Reticulin staining showed reticular fibers surrounding each tumor cell (original magnification × 100)

**Fig. 6:**
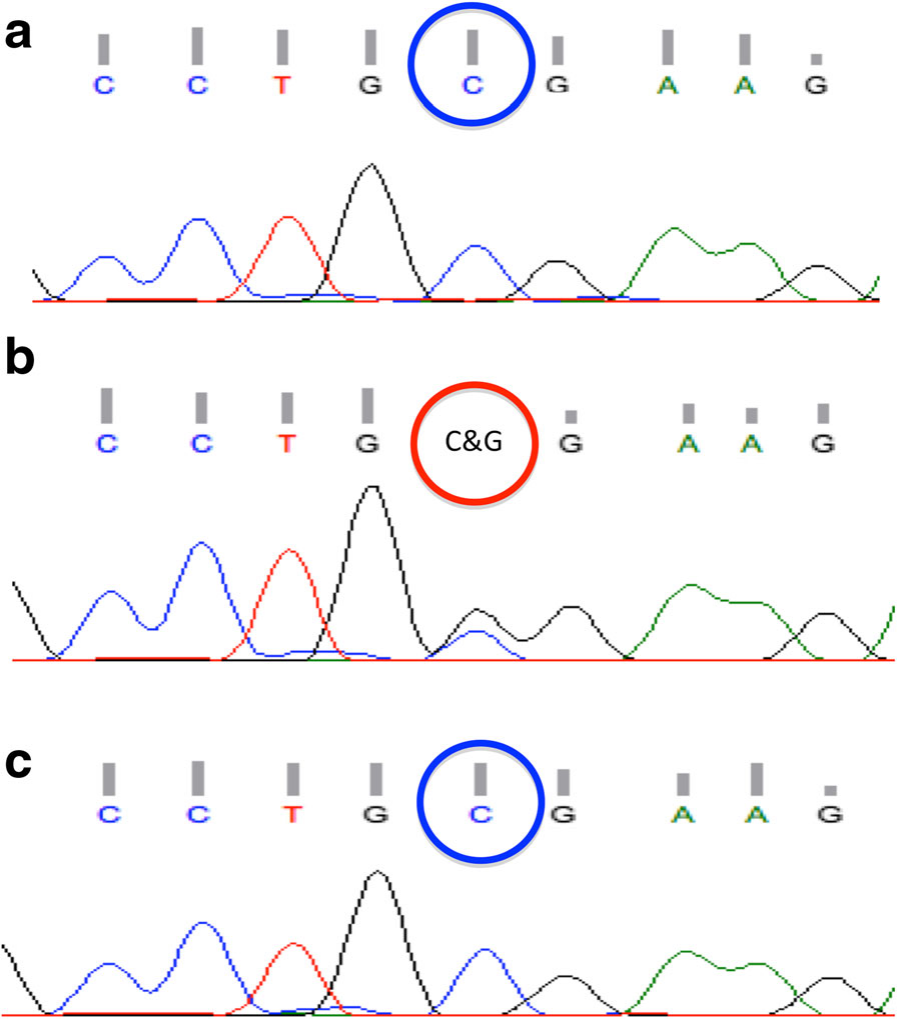
Analysis of a c.402C > G point mutation in the *FOXL2* gene in (**a**) the normal control (*negative; blue circle*), (**b**) the adult granulosa cell tumor patient used as a positive control (*positive; red circle*), and (**c**) our patient with rapid growth of mitotically active cellular fibroma (*negative; blue circle*)

Immunohistochemical staining was positive for vimentin, muscle-specific actin, alpha-smooth muscle actin, alpha-inhibin and progesterone receptor (Fig. 5a–e), and negative for calretinin, epithelial membrane antigen, desmin, estrogen receptor, CD10, CD99, chromogranin A, synaptophysin and tumor protein p53. The Ki-67/MIB-1 labeling index was 9.5 % (Fig. 5f). Reticulin staining showed reticular fibers surrounding each cell (Fig. 5g). This was not a typical feature of AGCT. We further analyzed genomic DNA (isolated from formalin-fixed, paraffin-embedded tumor tissue sections) for a *FOXL2* c.402C > G (p.C134W) point mutation that is observed in the majority of AGCT patients [18, 19]. The *FOXL2* mutation was detected in the AGCT case used as a positive control, but was absent from our case and the negative control (Fig. 6). Because necrosis and hemorrhage were not detected macro- or microscopically, a fibrosarcoma was ruled out. After performing the differential diagnoses outlined above, a final diagnosis of MACF was confirmed. Having obtained written informed consent, we evaluated the patient’s risk of recurrence. Based on our recommendation and the preference of the patient, we performed radical surgery (simple hysterectomy, left adnexectomy and omentectomy). The patient is undergoing regular follow-up. Recurrence of the tumor has not been observed in the 2-year postsurgical period.

## Discussion

MACF was first defined by Irving et al. [9] in 2006, based on a review of 75 cellular fibroma cases. The authors reported that the majority of MACF patients have a good clinical course compared to fibrosarcoma patients. However, there was a case where death was associated with peritoneal adhesion and capsule rupture and Irving et al. [9] could not necessarily conclude it to be a benign tumor. Therefore, pathological differential diagnoses of ovarian fibromatous tumors (including cellular fibroma, MACF and fibrosarcoma) are important since they influence decision-making, as well as, the prognosis of different treatment strategies.

Only six MACF cases have been reported since a definition of MACF was established in 2006 (Table 1). The natural history of MACF is still largely unknown. Five (83.3 %) of these recently reported cases were given a diagnosis of ovarian tumor. However, there has not been a single case where a diagnosis of MACF was confirmed preoperatively. Because there are no clinically useful serum tumor markers for MACF, or characteristic imaging findings, a preoperative diagnosis of MACF remains challenging at this time. There was a case report where the size of the tumor did not change during 10-years of follow-up [16]. In contrast, there was also a case report where a recurrent tumor, 8 cm in diameter, was detected in the pararectal region after 6 months of follow-up. In our case, a mass of 6 cm was found to have almost doubled in size, to 10 cm, during a 1-year follow-up period. Thus, when a solid ovarian tumor increases in size, even though malignancy findings are not found considering preoperative serum tumor marker values or imaging findings, MACF may well be included as a candidate for the differential diagnosis. In the 6 case reports published since the paper by Irving et al. [9], 5 patients (83.3 %) underwent open surgery, including a unilateral salpingo-oophorectomy in 2 patients [11, 13] and a radical operation (bilateral oophorectomy and hysterectomy) in 3 patients [12, 14, 15]. One patient treated with laparoscopic oophorectomy was reported by Yamada et al. [16] in 2015. However, because the follow-up period remains limited, the long-term prognosis has yet to be determined. No standard treatment for MACF currently exists, although overtreatment should be avoided in women requiring preservation of fecundity [2, 3]. Zong et al. [15] suggested that other risk factors, such as tumor size, growth rate and Ki-67 positivity, should be considered. As our case grew rapidly, the tumor size was relatively large, and the patient did not wish to conceive, we concluded that radical surgery was a reasonable option. Moreover, because there were no signs of recurrence, complete resection was not necessary for administering chemotherapy for the tumor associated with capsule rupture and peritoneal adhesion [15]. In contrast, there was a case where recurrence occurred 5-years after surgery [12]. Therefore, we believe long-term follow-up is necessary.

**Table 1:** Literature review of reported cases of mitotically active cellular fibroma

								Immunohistochemistry						
Author(s)	Age (y)	Size (cm)	PD	Rupture	Adhesion	Surgery	Mitoses (MF/10 HRP)	Ki-67 PI	Vimentin	SMA	α-inhibin	Calretinin	EMA	Prognosis
Kaku et al. [11]	32	6.6 × 6.0 × 4.4	OT	–	–	LSO	17	50	+	+	ND	ND	ND	12 month NED
Bucella et al. [12]	65	10.0 (First)	OT	–	–	TAH, BSO	4	9	+	ND	Focal+	ND	ND	Recurrence twice NED
		12.0 (Second)												
		8.0 (Third)												
Monteiro et al. [13]	13	19.0 × 15.0 × 12.0	OT	–	Omentum	RSO, OMT	5–7	ND	ND	ND	ND	ND	ND	36 month NED
Wu et al. [14]	76	9.0 × 6.0 × 5.0	PM	–	–	TAH, BSO	5–9	>10	+	ND	+	–	–	ND
Zong et al. [15]	39	10.0 × 7.0 × 4.0	OT	+	Left latum & uterus	TAH, LSO, OMT, AD, LD	3–5	10	+	+	ND	ND	–	66 month NED
Yamada et al. [16]	36	6.0	OT	ND	–	LS (RSO)	10	8.7	+	ND	Focal+	–	–	6 month NED
Present case	44	5.9 (First)	Myoma	–	–	LS (First), TAH, LSO, OMT (Second)	6.7	9.5	+	+	Focal+	–	–	26 month NED
		11.0 (Second)												

*Abbreviations: α* alpha, *AD* appendectomy, *BSO* bilateral salpingo-oophorectomy, *EMA* epithelial membrane antigen, *HRP* high power field, *LD* lymph node dissection, *LS* laparoscopic surgery, *LSO* left salpingo-oophorectomy, *MF* mitotic figure, *ND* not described, *NED* no evidence of disease, *OMT* omentectomy, *OT* ovarian tumor, *PD* preoperative diagnosis, *PI* proliferative index, *PM* pelvic mass, *RSO* right salpingo-oophorectomy, *SMA* smooth muscle actin, *TAH* total abdominal hysterectomy, *y* year, *+* positive, *−* negative

MACFs are defined as cellular fibromas with a high mitotic activity index (≥4 mitotic figures per 10 high-power fields) and mild to moderate nuclear atypia [10]. The differential diagnosis with fibrosarcoma is possible for nuclear atypia and the presence of necrosis and hemorrhage. There is a difficult case for the histological differential diagnosis of cellular fibroma and AGCT (especially the diffuse type) [20]. Cellular fibromas and AGCTs are represented by the sex cord-stromal tumors [1, 10]. However, the majority of borderline malignant sex cord-stromal tumors are AGCTs [21]. Therefore, the differential diagnosis with commonly benign cellular fibroma is important.

The *FOXL2* c.402C > G (p.C134W) point mutation is detected in 95.0 % of AGCT patients, especially those with the diffuse type [18–20]. It has been described that this point mutation does not reflect immunohistochemical staining pattern of FOXL2 [20]. In addition, the pattern of reticulin staining is different for these disease entities. Thus, combination of *FOXL2* point mutation analysis and reticulin staining can be employed in differential diagnosis of cellular fibroma and AGCT. In this case, we rejected a diagnosis of AGCT based on *FOXL2* mutation analysis and reticulin staining, and confirmed a final diagnosis of MACF.

## Conclusions

We report our experience of a case of rapid growth of a MACF of the ovary during a 1-year follow-up period. When preoperative serum tumor marker levels and/or imaging findings do not suggest ovarian cancer in instances of rapid growth of a solid ovarian mass, MACF should be considered as a differential diagnosis. Our case and review of the literature suggest that MACF of the ovary may represent a heterogeneous disease entity that requires an accumulation of case reports to establish diagnosis and treatment.

## Abbreviations

AGCT: Adult granulosa cell tumor
MACF: Mitotically active cellular fibroma
